# Long-term clinical outcomes of patients with nonsignificant transplanted renal artery stenosis

**DOI:** 10.1186/s12882-022-02691-0

**Published:** 2022-02-08

**Authors:** Manoela Linhares Machado Barteczko, Henry Campos Orellana, Gustavo Rocha Feitosa Santos, Attílio Galhardo, Gabriel Kanhouche, Ana Carolina Buso Faccinetto, Hélio Tedesco Júnior, José Osmar Medina Pestana, Ângelo Amato Vincenzo de Paola, Adriano Henrique Pereira Barbosa

**Affiliations:** 1 Department of Medicine, Cardiology Discipline, Federal University of Sao Paulo-UNIFESP, Sao Paulo Hospital, R. Napoleão de Barros, 715 - Vila Clementino, Sao Paulo/SP, 04024-002 Brazil; 2 Division of Nephrology, Hospital Do Rim E Hipertensao, UNIFESP, São Paulo/SP, Brazil

**Keywords:** Kidney transplantation, Transplant renal artery stenosis, TRAS, Nonsignificant stenosis, Angiography

## Abstract

**Background:**

Transplant renal artery stenosis (TRAS) is the main vascular complication of kidney transplantation. For research and treatment purposes, several authors consider critical renal artery stenosis to be greater than 50%, and percutaneous intervention is indicated in this scenario. However, there are no reports in the current literature on the evolution of patients with less than 50% stenosis.

**Method:**

This retrospective study included data from all patients who underwent kidney transplantation and were suspected of having TRAS after transplantation with stenosis under 50% independent of age and were referred for angiography at a single centre between January 2007 and December 2014.

**Results:**

During this period, 6,829 kidney transplants were performed at Hospital do Rim, 313 of whom had a clinical suspicion of TRAS, and 54 of whom presented no significant stenosis. The average age was 35.93 years old, the predominant sex was male, and most individuals (94.4%) underwent dialysis before transplantation. In most cases in this group, transplants occurred from a deceased donor (66.7%). The time between transplantation and angiography was less than one year in 79.6% of patients, and all presented nonsignificant TRAS. Creatinine levels, systolic blood pressure, diastolic blood pressure and glomerular filtration rate improved over the long term. The outcomes found were death and allograft loss.

**Conclusion:**

Age, sex and ethnic group of patients were factors that did not interfere with the frequency of renal artery stenosis. The outcomes showed that in the long term, most patients evolve well and have improved quality of life and kidney function, although there are cases of death and kidney loss.

## Background

Transplant renal artery stenosis (TRAS) is the main vascular complication of kidney transplantation [1]. TRAS usually occurs between the 3rd month and the 2nd year after transplantation, and the incidence varies from 1 to 23% depending on the diagnostic techniques and definitions used [2], although it can appear at any time, with refractory hypertension and/or dysfunction of the graft in the absence of rejection, ureteral obstruction or infection [3]. The factors attributed to TRAS may be perfusion of the clamp, incorrect suture technique or fibrotic inflammation due to the suture material [4], or graft rejection, cytomegalovirus (CMV) infection and the graft from a deceased donor [5, 6], the latter two being controversial factors in the literature [7].

TRAS diagnosis is carried out through several exams, as evaluating clinical parameters alone does not guarantee reliability when monitoring renal perfusion. Thus, it is necessary to evaluate clinical manifestations in addition to complementary exams, such as serum creatinine, refractory hypertension, Doppler ultrasonography (US Doppler), angiotomography and angioresonance [8].

This pathology is associated with a higher cardiovascular risk and increased mortality [9, 10]. For research and treatment purposes, several authors consider critical renal artery stenosis to be greater than 50% [11, 12], and percutaneous intervention is indicated in this scenario. However, there are no reports in the current literature on the evolution of patients with less than 50% stenosis.

## Methods

### Patient selection and study design

This was a retrospective study approved by the local research ethics committee. Between January 2007 and December 2014, 6,829 kidney transplants were performed at Hospital do Rim. Patients with suspected TRAS due to refractory hypertension, renal dysfunction and/or increased PSV above 200 cm/s were referred for angiography. Patients with less than 50% stenosis were followed for a long period. Patients lost to follow-up were excluded from the analysis. The mean follow-up time of the patients was 8.5 years (5–12).

### Data acquisition

Demographic and clinical data were collected from medical records. Procedure data were collected from our lab database. We used REDCap electronic data capture tools hosted at HSP – UNIFESP (13, 14). REDCap (Research Electronic Data Capture) is a secure, web-based software platform designed to support data capture for research studies, providing 1) an intuitive interface for validated data capture; 2) audit trails for tracking data manipulation and export procedures; 3) automated export procedures for seamless data downloads to common statistical packages; and 4) procedures for data integration and interoperability with external sources.

### Study endpoints

Primary outcomes were defined as all-cause mortality and allograft survival. Allograft loss was defined by the need for permanent dialysis, as documented by the renal transplant team notes.

Secondary outcomes were defined as serum creatinine (SCr), estimated glomerular filtration rate (eGFR) by Cockcroft-Gault, systolic blood pressure (SBP), and diastolic blood pressure (DBP) at 1 month and 1 year postarteriography.

Primary outcomes were defined as all-cause mortality and allograft survival from renal angiography. Allograft loss was defined by the need for permanent dialysis as documented by the renal transplant team notes.

Secondary outcomes were defined as serum creatinine (SCr), estimated glomerular filtration rate (eGFR) by Cockcroft-Gault, systolic blood pressure (SBP), and diastolic blood pressure (DBP) at 1 month and 1 year postarteriography.

### Statistical analysis

We used multiple imputations (*mice* package in R) to handle missing values (MVs). We used a predictive mean matching model for numeric variables, logistic regression (logreg) for binary variables (with 2 levels) and Bayesian polytomous regression (polyreg) for factor variables (> = 2 levels). We did not impute missing values for the clinical outcomes.

Normally distributed data are presented as the mean ± SD, and skewed data are presented as the median [interquartile range (IQR)]. Normality of distribution and variances were checked using histograms, Kolmogorov-Smirnoff tests, normal probability plots and residual scatter plots. Chi-square, Mann–Whitney, or two-tailed t tests were used for comparisons of baseline data.

Statistical analyses included a series of logistic regression models to predict the combined endpoint of death, kidney loss or retransplantation as the main endpoint, using the odds ratio and 95% confidence intervals to estimate relative risk. Our regression models were built using a stepwise approach, limiting to 2 to 3 variables per step or per model.

We selected the variables with the highest partial R^2^ for the respective outcomes among those with high collinearity (intervariable R^2^ > 0.25 or variance inflation factor > 10). *P* values < 0.05 were considered statistically significant. Analyses were carried out using R [v4.0.0].

## Results

### Patient characteristics

A total of 6,829 kidney transplants were performed at Hospital do Rim; 313 of them had clinical suspicion of TRAS, and 54 patients had no significant stenosis (Table 1). The age of this group ranged from 14 to 68 years old. The predominant sex was male; most individuals were submitted to dialysis before transplantation with an average time greater than 2 years. The diagnosis of hypertension was found in most of the patients, and all of these patients used medication for control and treatment, with 35% using two associated medications. The most common CKDs were diabetic nephropathy and glomerulonephritis. Transplants occurred mostly from a deceased donor, and only one of these was undergoing retransplantation.

**Table 1:** Patient characteristics

N	54
Age (years) (mean (SD))	35.93 (15.96)
Sex (%, women)	10 (18.5)
Whites (%)	22 (40.7)
Weight (kg, D0) (mean (SD))	64.61 (18.32)
Height (cm, D0) (mean (SD))	168.50 (11.39)
BMI (kg/m2) (mean (SD))	22.39 (4.83)
Time on dialysis (months) (mean (SD))	33.10 (25.88)
Type of dialysis (%)	
Haemodialysis	49 (96.1)
Peritoneal dialysis	1 (2.0)
Conservative treatment	1 (2.0)
HLA class (%)	
Ident (I)	27 (54.0)
Hapto (II)	6 (12.0)
Dist (III)	6 (12.0)
CAD	11 (22.0)
Hypertension (%)	43 (79.6)
Diabetes mellitus (%)	15 (27.8)
Dyslipidaemia (%)	5 (9.3)
Smoking (%)	3 (5.6)
EBV serum (% positive)	5 (13.9)
CMV serum (% positive)	47 (90.4)
CMV prophylaxis (%)	17 (32.1)
Prior hypertensive nephropathy (%)	9 (16.7)
Prior diabetic nephropathy (%)	12 (22.2)
Prior polycystic nephropathy (%)	3 (5.6)
Prior glomerulonephritis (%)	10 (18.5)
Prior other diagnoses (%)	6 (11.1)
Prior unknown cause of CKD (%)	19 (35.2)
Other diseases (%)	
NA	49 (90.7)
Indeterminate	1 (1.9)
Repeated urinary infection	1 (1.9)
Renal malformation	1 (1.9)
Repeating pyelonephritis	1 (1.9)
Posterior urethral valve	1 (1.9)
Alive donor (%)	18 (33.3)
Deceased donor (%)	36 (66.7)
Panel (%) (mean (SD))	7.23 (18.69)
Second transplantation (index Tx)	1 (1.9)
Time since transplantation (months) (median [IQR])	5.00 [3.00, 9.00]
TAV (median [IQR])	30.00 [25.00, 37.00]
TIF (mean (SD))	17.67 (12.14)
Total cholesterol (mg/dL) (mean (SD))	170.93 (46.93)
HDL-C (mg/dL) (mean (SD))	43.85 (11.50)
LDL-C (mg/dL) (mean (SD))	106.45 (46.22)
Cause of death (donor) (%)	
NA	19 (35.2)
CV disease	5 (9.3)
Neurologic disease	17 (31.5)
Trauma	13 (24.1)
Type of graft (%)	
NA	14 (25.9)
Gregoir	32 (59.3)
Politano	4 (7.4)
Ureteropielo	4 (7.4)
Blood type (%)	
NA	7 (13.0)
A	15 (27.8)
AB	3 (5.6)
B	7 (13.0)
O	22 (40.7)
RH factor = + (%) (n = 16)	16 (100.0)
Blood transfusion	17 (33.3)
Baseline Meds	
Anti-hypertensive meds at baseline (%)	43 (100.0)
ACEi at baseline (%)	17 (31.5)
ARB at baseline (%)	6 (11.1)
Beta-blocker at baseline (%)	21 (38.9)
Diuretic at baseline (%)	10 (18.5)
Vasodilator at baseline (%)	14 (25.9)
Calcium channel blockers at baseline (%)	25 (46.3)
Central alpha-antagonist at baseline (%)	5 (9.3)
Insulin at baseline (%)	10 (18.5)
Oral hypoglycaemic meds at baseline (%)	5 (9.3)
Hypolipidaemic meds at baseline (%)	3 (5.6)
Simvastatin at baseline (%)	2 (3.7)
Atorvastatin at baseline (%)	0 (0)
Rosuvastatin at baseline (%)	0 (0)
Post-Tx smoking	2 (3.9)
Treatment for rejection	18 (34.6)
ISS tacrolimus	36 (70.6)
ISS mycophenolate	18 (35.3)
ISS prednisone	50 (98.0)
ISS cyclosporine	15 (29.4)
ISS azathioprine	34 (66.7)

The patients in this study mostly used the following immunosuppressive regimens: tacrolimus, azathiopine and prednisone, with the use of prednisone appearing in all patients in the study.

### Angiographic data

The time between transplantation and angiography was less than one year in 79.6% of patients, and of the 54 studied, all presented nonsignificant TRAS, and all were submitted to US Doppler. Of these patients, only 3.7% had PSV values within normal parameters of up to 200 cm/s. The patients who underwent Angio TC, Angio RM, Angio 2D and Angio 3D are shown in Table 2. Most patients have a degree of stenosis of 30%.

**Table 2:** Doppler/angiography measures

Suspected TRAS	53 (100.0)
US Doppler	52 (100.0)
A_angio_TC (mean (SD))	28.20 (13.86)
Stenosis A 3D (median [IQR])	30.00 [30.00, 30.00]
Luminal reduction (%) (mean (SD))	29.35 (7.69)
Translesional gradient (mean (SD))	11.31 (5.76)
Stenosis rate (mean (SD))	30.06 (9.13)
Angio TC	15 (31.2)
Angio RM	0 (0)
Angio 2D	48 (96.0)
Angio 3D	26 (52.0)
Any stenosis (%)	54 (100.0)
Stenosis ≥ 30% & < 50% (%)	40 (74.1)
Stenosis at iliac aa	1 (1.9)
Stenosis at renal artery ostium	31 (57.4)
Stenosis at RA body	22 (40.7)
Stenosis at RA branches (%)	0 (0)
Stenosis at polar aa (%)	0 (0)
Stenosis type (concentric) (%)	8 (14.8)
Stenosis type (eccentric) (%)	0 (0)
Stenosis type (diffuse) (%)	1 (1.9)

### Laboratory tests and parameters

SCr improved over a year when comparing creatinine before angiography, and after one year of angiography, 11% more patients had normal values (Table 3).

**Table 3:** Substitute outcomes (follow-up lab parameters)

Creatinine at baseline (mg/dL) (median [IQR])	1.68 [1.44, 2.24]
Creatinine at 1 month (mg/dL) (median [IQR])	1.73 [1.43, 2.19]
Creatinine at 1 year (mg/dL) (median [IQR])	1.56 [1.31, 2.00]
Delta creatinine at 1 month (mg/dL) (median [IQR])	0.05 [-0.22, 0.18]
Delta creatinine at 1 year (mg/dL) (median [IQR])	-0.18 [-0.39, 0.10]
SBP prearteriography (mmHg) (mean (SD))	144.00 (22.98)
SBP at 1 month (mmHg) (mean (SD))	136.98 (18.74)
SBP at 1 year (mmHg) (median [IQR])	130.00 [120.00, 140.00]
Delta SBP at 1 month (mmHg) (mean (SD))	-7.34 (25.42)
Delta SBP at 1 year (mmHg) (mean (SD))	-9.89 (34.45)
DBP prearteriography (mmHg) (mean (SD))	86.88 (15.00)
DBP at 1 month (mmHg) (mean (SD))	81.94 (10.80)
DBP at 1 year (mmHg) (mean (SD))	80.44 (10.50)
Delta DBP at 1 month (mmHg) (mean (SD))	-4.86 (17.11)
Delta DBP at 1 year (mmHg) (mean (SD))	-5.18 (18.04)
VPS (mean (SD))	395.10 (113.02)
VPS post (median [IQR])	256.50 [137.75, 297.50]
Delta VPS (mean (SD))	-207.70 (171.75)
GFR at baseline (mean (SD))	44.92 (31.94)
GFR at 1 month (mean (SD))	44.92 (28.92)
GFR at 1 year (mean (SD))	48.50 (31.98)
Change in GFR at 1 month (median [IQR])	0.00 [-5.00, 2.00]
Change in GFR at 1 year (median [IQR])	0.50 [-2.75, 10.00]
Drop > 0.1 mg/dL in creatinine at 1 month	29 (53.7)
Drop > 0.1 mg/dL in creatinine at 1 year	29 (53.7)
Any drop in SBP at 1 month	27 (50.0)
Any drop in SBP at 1 year	21 (38.9)
Any drop in DBP at 1 month	23 (42.6)
Any drop in DBP at 1 year	21 (38.9)
Any drop in VPS	13 (24.1)

In the case of SBP, their average values over a year were improved. In addition, one month and one year after angiography, a great number of patients presented a drop in SBP values. Diastolic blood pressure had the same improvement over time (Table 3).

For the eGFR, an improvement was noted one month after angiography, but after one year, there was an increase in these values, and fewer patients had glomerular filtration values within the normal range.

### Mild clinical outcomes

There was a general increase in the number of patients using medications to treat hypertension before and after angiography. However, some of the patients experienced a decrease in the amount of medication they used (Table 4).

**Table 4:** Soft clinical outcomes

Suspected restenosis	1 (1.9)
Clinical follow-up	50 (96.2)
Anti-hypertensive meds pre (mean (SD))Table 3	2.28 (1.01)
Anti-hypertensive meds post (mean (SD))	2.16 (1.08)
Absolute change in anti-hypertensive meds (mean (SD))	0.05 (0.99)
Any drop in anti-hypertensive meds	8 (14.8)

### Clinical outcomes

The outcomes presented were allograft loss and death, all due to cardiovascular causes. Retransplantation was not found in any of the cases, and the remainder had no long-term outcome (Table 5).

**Table 5:** Hard clinical outcomes

New graft (new transplantation)	0 (0)
Allograft loss	12 (22.2)
Death	4 (7.4)
CV death	4 (7.4)
Compound outcome	16 (29.6)

Of the patients who died, all were male, with an average age of 53.5 years old. All were submitted to haemodialysis. Seventy-five percent had a diagnosis of hypertension using medication. Fifty percent of them had diabetic nephropathy as the main underlying disease. All were recipients from deceased donors. The immunosuppressive regimen was tacrolimus, mycophenolate, prednisone or cyclosporin, azathiopine, and prednisone.

All of them had suspected TRAS and underwent US Doppler with 100% of the PSV values above 300 cm/s. The degree of stenosis was in the 40% range in 50% of the individuals, and the places of stenosis were in the renal artery ostium in 50% of the patients and in the renal artery body in 50%. Only 25% underwent a new US Doppler after angiography, with a PSV value above 300 cm/s. All patients continued to use medication to control and treat their hypertension after angiography. The majority of patients presented better SBP, DBP, creatinine levels and glomerular filtration rates over one year.

Of the patients who presented allograft loss outcomes, 81.8% were male, with an average age of 34.2 years old. A total of 100% of these patients were undergoing haemodialysis. The underlying disease found in the majority was glomerulonephritis. A total of 90.9% of patients reported a positive diagnosis of hypertension, and 100% of them used medication to control and treat the disease. The type of donor was mostly deceased donors (90.1%), and the causes of death were 50% trauma, 30% neurological disease and 10% cardiovascular disease. The ISS scheme of the majority was tacrolimus, mycophenolate, and prednisone. In this group, 100% of patients had suspected TRAS, as well as Doppler ultrasonography, and only 2 patients had PSV within the normal value, that is, up to 200 cm/s. Most had at least a 30% degree of stenosis. A total of 36.4% of patients underwent new US Doppler, and all PSVs remained within normal values. All patients used medication to control hypertension after transplantation and angiography. When we analysed the amount of medication used to treat hypertension before and after angiography, as used by this group of patients, we noticed that there was a decrease in the number of associations. Before, it was well known that patients used two or three combinations of drugs, and after angiography, this value dropped to one or two associations. In this group, only four patients showed improvement in creatinine levels. SBP, DBP and glomerular filtration rate also improved in a few patients.

## Discussion

TRAS is the main vascular complication of kidney transplantation. Controversies in the literature about the factors that trigger stenosis in the artery of the transplanted kidney are numerous, and among them we can find type of donor, time between transplant and stenosis, and even the technique used for arterial anastomosis [13]. In 2015, a study showed that the association of the first lesions with complications of the surgical technique and of the graft is related to the pathophysiology and temporality of the lesions, so that for patients with renal graft, TRAS becomes an important vascular complication, as indicated by risk factors and clinical signs, such as worsening renal function, stenosis, increase in antihypertensive drugs, and high PSV value, among others [14, 15]. In this study, 54 patients were analysed, and all of them presented nonsignificant stenosis.

Previous studies of patients with significant stenosis showed that there was no relationship between age and the degree of stenosis [16, 17]. The average age found was 55 years in one study and 37 years in the other. In this study, an average age of 35.93 years was found. However, these previous studies showed that the patients’ sex was mostly male, corroborating the present study in which the majority (81.5%) of the individuals were also male [16–18]. Dialysis is recommended for patients with end-stage renal disease (ESKD) [19], which was the case in this study, in which the entire number of patients was submitted to some type of dialysis or conservative treatment, corroborating the findings.

In studies with significant stenosis, a diagnosis of systemic arterial hypertension was observed, despite the use of medications being superior to three associated types. The systolic averages found in these studies were 170 ± 30 mmHg and diastolic 105 ± 15 mmHg. After follow-up and endovascular treatment, there was an improvement in pressure, and the averages became 120 ± 20 mmHg for systolic and 75 ± 15 mm Hg for diastolic, with decreases in up to two associated medications [16]. The averages in the patients in this study were considerably lower than the averages in the patients in studies with significant stenosis. Before and after angiography, the highest number of associated medications was two, and in the intervals before and after angiography, this value increased by 6%. After transplantation, several conditions and aetiologies exist for the onset or worsening of SAH, such as toxicity of immunosuppressive drugs, graft rejection, and recurrence of the original kidney disease. Among these conditions is stenosis of the renal artery, which is responsible for hypertension in 10% of transplant recipients but has great potential for cure [20–26].

The Brazilian Society of Nephrology names glomerulonephritis (23.5%), hypertensive nephrosclerosis (24.1%) and diabetes mellitus (16.6%) as the main causes of chronic renal failure (16.6%) [19, 27]. Relating these statistics to this study, CKD categorized as indeterminate occurred in more than 35% of the studied patients, followed by glomerulonephritis (16.7%), diabetic nephropathy (14.8%) and hypertensive nephropathy (7.1%).

The transplanted organs are of various origins, and according to Associação Brasileira de Transplante de Órgãos (ABTO), on average, 59% of transplants come from living donors and 41% from deceased [27, 28]. In 1998, in a study with 676 kidney transplants, Lopes et al*. *[29] reported an index of 1.63% of stenosis and that all the incidences of stenosis occurred in deceased donor transplants, while in the study by Mendes et al., most recipients received a donation from a living donor. In the present study, with a nonsignificant TRAS, the donor type was deceased donors in 66.7% of the evaluated cases. During the statistical analysis, it was found that there was no relationship between the type of donor and the condition of the patient with significant or nonsignificant stenosis of the renal artery. The tendency towards a lower number of stenoses when using a deceased donor may be attributed to the more frequent use of aortic patches [16].

In a study published by Medina [30] in 2017, it was observed that cyclosporine was replaced by tacrolimus and azathiopine by mycophenolate over the years of his research and revealed that in the first years, the combination of cyclosporine with azathiopine and prednisone was predominant. However, the use of tacrolimus has increased over time, and an association with azathiopine was found at a higher percentage than the association with mycophenolate. This finding corroborates this study, in which all immunosuppressive associations were observed with prednisone, and drug combinations involving tacrolimus were more commonly used in patients than those involving cyclosporine. In the choice between azathiopine and mycophenolate to associate with other immunosuppressive agents, azathiopine appears in a greater number of patients, regardless of the association. In this study, the most common association of immunosuppression was 37% of kidney transplant recipients with tacrolimus, azathiopine and prednisone. Patients with high immunological risks and retransplants mainly use the scheme involving tacrolimus, mycophenolate and prednisone due to the possible reduction observed in the incidence of treated acute rejection [31]. Despite the use of these immunosuppressants to decrease the incidence of treated acute rejection, it should also be taken into account that this scheme improves patient and graft survival [32, 33]. In this study, when we analysed the patients who presented the outcome of allograft loss, it was noted that their immunosuppressive regimens were mostly tacrolimus, mycophenolate and prednisone, while the patients who presented the outcome died mostly using other immunosuppressive regimens.

The time between transplantation and angiography was less than a year, as in other studies, but with patients with significant stenosis, there was no relationship between the time and the degree of stenosis [7, 13].

The Doppler echo exam is chosen for recipients with graft dysfunction, and the increase in peak velocity suggests that the vascular flow is compromised and that when stenosis is suspected, it is necessary to perform angiography [20, 34, 35]. Thus, the gold standard for definitively diagnosing stenosis is angiography, as it confirms the lesion that ultrasound has identified; thus, it is possible to plan the therapeutic approach and ascertain the need for intervention [11, 21, 34, 35]. In this study, all patients underwent Doppler examination and had suspected stenosis, and after angiography, a nonsignificant stenosis < 50% was suggestive.

To diagnose TRAS, the cut-off values are not homogeneous in the literature, with the most consensual values for direct parameters being PSV values > 180–200 cm/s [36–38]. In this study, the PSV values were considered normal up to 200 cm/s. Only two out of 54 subjects had PSV within normal values prior to angiography. Of the patients who had high PSV, the highest percentage was in the range of 201 to 400 cm/s, considering that three patients studied had PSV greater than 601 cm/s. This shows that despite a high PSV, patients with nonsignificant stenosis had PSVs closer to normal levels. After angiography, 20 patients underwent a new Doppler ultrasonography, seven of whom had SPV within normal limits. In the United States, in clinical practice, it is common to use CT angiography and MRI angiography, whereas in Europe, these methods are used only when, after renal Doppler, doubts about the diagnosis persist or when there are strong hypotheses, such as patients with multiple risk factors, taking into account all contraindications inherent to these procedures [22, 34–37]. In this study, angio-CT was performed in less than 40% of patients, while angio-MRI was not performed.

Stenosis is considered significant when it compromises more than 50% of the arterial lumen, and the therapeutic approach to treatment depends on the degree of stenosis and its location. In cases of mild stenosis, that is, cases where blood pressure is controllable with medication and the creatinine level remains stable and < 3 mg/dl, conservative treatment is commonly used [11, 12]. After evaluating and performing tests such as US Doppler and angio-CT, among others, it was found that the degree of stenosis in this study ranged from 10 to 46%; therefore, there were no significant degrees of stenosis, and these patients did not undergo intervention.

Renal graft dysfunction of vascular aetiology is usually secondary to stenosis of the transplanted renal artery. However, high levels of serum creatinine and hypertension may also be present in patients with stenosis [39]. In these patients, creatinine levels returned to values considered normal for a renal transplant patient, that is, values at the maximum limit of normality or slightly increased. CKD can be classified according to the glomerular filtration rate into five stages [28]. Other parallel studies are unanimous in showing that a glomerular filtration value > 90 ml/min/1.73 m^2^ is the best parameter associated with prolonged organ survival [40–42]. Renal function should be monitored using the glomerular filtration rate estimated by the Cockroft-Gault equation [43–46]. In these cases, the measurement of serum creatinine is not recommended because there is no linear relationship between the plasma creatinine level and glomerular filtration rate [45, 46]. Some studies of converting the therapeutic regimen of cyclosporine and azathiopine to tacrolimus and mycophenolate or the use of mycophenolate and the reduction of cyclosporine doses have shown significant improvements in the glomerular filtration rate [47–51]. These data are in accordance with this study since more patients used the tacrolimus and mycophenolate regimen and showed an improvement in the glomerular filtration rate.

The outcomes found in this study varied mainly between allograft loss and death, while the other patients continued to evolve well with transplantation and angiography. Comparing outcome, death and allograft loss, it was found that the average age of patients who died was high compared to those who had kidney loss. One study [52] showed that the average age of patients who died after kidney transplantation was over 40 years old and that death after transplantation occurred in 10.6% of the studied patients. The incidence of kidney loss among the patients was 20.9%. These data corroborate the study showing that the death rate for patients undergoing transplantation is relatively low and that the age of these patients is over 40 years old. However, when the average survival time of these individuals was evaluated, in the study previously mentioned, it was 14.4 months, while in the present study, the survival time was much longer.

The percentage of patients with allograft loss was significant in the study mentioned and in the present study, at 20.7%, and the average age of these patients was over 30 years old.

There were a total of 4 deceased recipients, and all were male. The majority were white, and all patients from these groups underwent haemodialysis before transplantation. Fifty percent presented with underlying diabetic nephropathy. All donors in this group were also deceased, and the causes of death were mostly neurological disease (3) and trauma (1). One hundred percent were submitted to US Doppler and were confirmed to have stenosis to any degree.

Recipients with organ loss were mostly male (81.2%) and dark-skinned (45.4%). One hundred percent were submitted to haemodialysis before the transplant. The underlying diseases found were glomerulonephritis, diabetic nephropathy and indeterminate in 45.4% of patients. The majority were deceased donors, and the causes of death were trauma, neurological disease and cardiovascular disease. In 45.4% of the patients, rejection was declared, and treatment was necessary. All patients underwent US Doppler and presented any stenosis degree.

In conclusion, age, sex and ethnic group of patients are factors that did not interfere with the frequency of renal artery stenosis. The outcomes showed that in the long term, death occurs in older patients with the outcome of allograft loss. Even so, most patients progress well and have improved quality of life and kidney function.

It was not possible to establish significant associations between nonsignificant stenosis and factors such as DM, SAH and other underlying diseases in this study, and it was not possible to associate EART < 50% with graft type, time between transplant and angiography, degree of stenosis, time on dialysis or its type, SPV values, levels of creatinine or glomerular filtration rate, SBP and DBP. Thus, further studies are necessary in this scope because there is no previous literature on nonsignificant stenosis.

In addition, this study has limitations because it is a retrospective study. There are no previous studies on patients with nonsignificant stenosis, and the search was performed in a single centre.

## Data Availability

All data generated or analysed during this study are included in this published article.

## Abbreviations

BMS: Bare metal stent
CMV: Cytomegalovirus
CIT: Cold ischaemia time
DBP: Diastolic blood pressure
DES: Drug-eluting stent
DGF: Delayed graft function
DM: Diabetes mellitus
eGFR: Estimated glomerular filtration rate
ESRD: End-stage renal disease
PTA: Percutaneous transluminal angioplasty
PSV: Peak systolic velocity
SBP: Systolic blood pressure
SCr: Serum creatinine
TRAS: Transplant renal artery stenosis
US: Ultrasonography
